# Radiation-related occupational cancer and its recognition criteria in South Korea

**DOI:** 10.1186/s40557-018-0219-y

**Published:** 2018-02-02

**Authors:** Songwon Seo, Dalnim Lee, Ki Moon Seong, Sunhoo Park, Soo-Geun Kim, Jong-Uk Won, Young Woo Jin

**Affiliations:** 1 National Radiation Emergency Medical Center, Korea Institute of Radiological & Medical Sciences, 75, Nowon-ro, Nowon-gu, Seoul, 01812 Republic of Korea; 2 Department of Preventive Medicine, Korea University College of Medicine, Seoul, Korea; 3 Department of Occupational Medicine, Sungkyunkwan University, School of Medicine, Seoul, Korea; 4 The Institute for Occupational Health, Yonsei University College of Medicine, Seoul, Korea

**Keywords:** Radiation exposure, Occupational cancer, Recognition, Korea

## Abstract

Ionizing radiation is a well-known carcinogen, and is listed as one carcinogenic agent of occupational cancer. Given the increase in the number of workers exposed to radiation, as well as the increase in concern regarding occupational cancer, the number of radiation-related occupational cancer claims is expected to increase. Unlike exposure assessment of other carcinogenic agents in the workplace, such as asbestos and benzene, radiation exposure is usually assessed on an individual basis with personal dosimeters, which makes it feasible to assess whether a worker’s cancer occurrence is associated with their individual exposure. However, given the absence of a threshold dose for cancer initiation, it remains difficult to identify radiation exposure as the root cause of occupational cancer. Moreover, the association between cancer and radiation exposure in the workplace has not been clearly established due to a lack of scientific evidence. Therefore, criteria for the recognition of radiation-related occupational cancer should be carefully reviewed and updated with new scientific evidence and social consensus. The current criteria in Korea are valid in terms of eligible radiogenic cancer sites, adequate latent period, assessment of radiation exposure, and probability of causation. However, reducing uncertainty with respect to the determination of causation between exposure and cancer and developing more specific criteria that considers mixed exposure to radiation and other carcinogenic agents remains an important open question.

## Background

Ionizing radiation is classified as a Group 1 carcinogen in humans by the International Agency for Research on Cancer (IARC), and is listed as one carcinogenic agent of occupational cancer by the International Labor Organization (ILO) and the Enforcement Decree of the Labor Standards Act in Korea [1–3]. Ionizing radiation is called “radiation” henceforth in this paper. Radiation is utilized for various purposes, and both the number of radiation-related facilities and the number of radiation workers have also increased by about 10 and 4% per year, respectively [4]. Radiation exposure has been well managed under 5% of the occupational dose limit which is a 100 mSv in 5 years with a maximum of 50 mSv in any single year, in most radiation workers in Korea. However, some occupations, such as workers who perform non-destructive testing (NDT) and radiologists, are exposed to relatively higher radiation levels than other radiation-related occupations [5]. Moreover, due to an increase in social concerns about occupational cancer, the number of occupational cancer claims related to radiation exposure is increasing, especially among semiconductor manufacturing and NDT workers. In general, criteria for the recognition of radiation-related occupational cancer are based on the type of cancer, exposure assessment, probability of causation, and general principles of compensation for occupational diseases. These criteria should be updated with new scientific evidence and social consensus. The aim of this study, therefore, was to review the recognition criteria for radiation-related occupational cancer and identify the characteristics of radiation exposure and diagnosed cases in the workplace in Korea. This review provides a comprehensive reference for understanding criteria for the recognition of radiation-related occupational cancer, which can help to guide reasonable and scientific decision making.

## Review

### Occupational exposure in Korea

Exposure assessment is essential for identifying whether cancer incidence among workers is caused by harmful agents in the workplace. In Korea, depending on the occupation type, radiation exposure in individual workers has been monitored and managed by two government institutes, the Nuclear Safety and Security Commission (NSSC) and the Centers for Disease Control and Prevention (CDC), with their own National Dose Registries (NDR). To determine whether cancer occurrence in the workplace is associated with radiation exposure, these NDRs are investigated first for radiation exposure assessment. Radiation workers in the NDR who are managed by the NSSC are grouped into nine categories: public institution, educational institution, non-destructive industry, industrial organization, research institute, nuclear power plant, medical institution (except for workers using diagnostic x-ray generators), military, and production and sales [6]. Since the NDR was started in 1984, the average exposure dose for radiation workers has been in steady decline to nearly 1 mSv per year or less, except for NDT workers, whose exposure levels were the highest with average doses of 2.37–3.87 mSv/year in the recent five years (Table 1) [5, 6]. Exposure doses of diagnostic radiation workers, who mainly work with x-ray generators in hospitals, were managed by the CDC’s NDR and grouped into five categories: radiologic technologists, physicians, dentists, dental hygienists, and other radiation workers [7]. Exposure doses have been in steady decline over the last 10 years among diagnostic radiation workers. Exposure levels were highest among radiologic technologists, with average doses of 0.85–1.21 mSv/year in the recent 5 years (Table 1) [8].

**Table 1:** Number of workers and exposure dose (mSv) according to occupation type in Korea

Year		2010		2011		2012		2013		2014	
Category		Number of workers	Mean dose	Number of workers	Mean dose	Number of workers	Mean dose	Number of workers	Mean dose	Number of workers	Mean dose
Radiation workers	Medical institutes	3833	0.99	4133	0.96	4376	0.87	4734	0.73	5038	0.55
	Industry	5464	0.10	5456	0.03	6352	0.07	5842	0.16	5237	0.02
	NDT	5852	2.43	6075	2.39	6792	3.43	7166	3.87	7530	2.37
	Production and sales	1243	0.67	1573	0.53	1563	0.85	1702	0.41	1912	0.29
	Research institutes	2062	0.07	2139	0.05	2232	0.03	2198	0.03	2183	0.02
	Educational institutes	4876	0.05	4954	0.05	4816	0.04	4788	0.04	4521	0.06
	Public institutes	466	0.02	827	0.61	872	0.57	932	0.42	961	0.41
	Military	236	0.05	241	1.81	264	0.02	280	0.03	264	0.08
	Nuclear power plant	13,236	1.20	14,758	0.80	15,023	0.73	14,780	0.82	14,253	0.58
	Total	37,268	0.96	40,156	0.81	42,290	0.96	42,422	1.07	41,899	0.72
Diagnostic radiation workers	Radiation technologist	18,722	1.21	19,791	1.16	20,523	1.01	21,636	0.94	22,419	0.85
	Physician	11,661	0.34	12,622	0.36	13,076	0.32	13,738	0.32	14,950	0.31
	Dentist	12,822	0.16	13,849	0.18	14,424	0.15	14,905	0.15	15,951	0.15
	Dental hygienist	6110	0.13	7088	0.15	7727	0.12	8064	0.12	8912	0.12
	Diagnostic radiologist	1468	0.41	1545	0.29	1456	0.32	1448	0.31	1475	0.24
	Nurse	2177	0.4	2936	0.37	3171	0.33	3397	0.32	4891	0.22
	Nursing assistant	817	0.3	927	0.26	873	0.24	846	0.3	1081	0.19
	Medical assistant	161	0.3	198	0.34	168	0.55	222	0.49	329	0.34
	Others	1676	0.47	1474	0.42	1517	0.33	1676	0.68	1088	0.34
	Total	55,614	0.58	60,430	0.56	62,935	0.48	65,932	0.47	71,096	0.41

Source: 2015 Nuclear Safety yearbook [5] and 2014 Occupational Radiation Exposure in Diagnostic Radiology in Korea [8]

*NDT* non-destructive testing

### Radiation carcinogenesis

The initial mechanism of radiation-induced cancer is not different from the mechanisms of other harmful agents, such as toxic chemicals and ultraviolet radiation, in terms of DNA damage. It is well-known that many innate defense mechanisms against radiation damage occur in various ways (e.g., removal of oxidative stress and damaged cells, DNA repair) in the human body, and damaged cells or DNA that remain may cause tissue or organ dysfunction and malignant disease such as cancer and heritable disease. In general, health risks from radiation exposure are classified into two groups: tissue reactions, which are conventionally referred to as deterministic effects, and stochastic effects. Tissue reaction effects include organ malfunction such as skin burns, bone marrow failure, and intestinal damage, which occur above a threshold dose below which there is no increased risk and are considered non-cancer damaging effects. In contrast, stochastic effects are assumed to have no threshold dose and occur by chance, with the probability of the effect increasing as exposure dose increases. The main risks associated with stochastic effects are cancer and genetic defects, and generally occur 1–2 years after exposure for leukemia and 5–10 years after exposure for solid cancer. Thus, radiation-related occupational cancer can be considered a stochastic effect of radiation exposure.

The IARC and the U.S. National Toxicology Program (NTP) classify radiation (commonly referred to as ionizing radiation), including x-rays and gamma rays, as “Group 1” and “Known” carcinogens, respectively, according to their own classification criteria [9]. The European Agency for Safety and Health at Work similarly interprets radiation carcinogenesis according to the classification of carcinogens, mutagens, and reprotoxicants (CMR) substances, based largely on human evidence [10]. Regarding the evaluation of a causal association between radiation exposure (i.e., x-ray and gamma rays) for individual cancer (organ) sites, the IARC has categorized cancer sites into “strong evidence” and “potentially having limited or inadequate evidence” based on up-to-date scientific evidence [9]. Cancer sites with “strong evidence” are listed in Table 2, and these evaluations were carried out based on biological data and epidemiological evidence.

**Table 2:** Cancer sites/ tumors with sufficient evidence for causal associations with x-ray and gamma-ray exposure

Organ site	Selected key studies
Stomach	Boice et al. (1988) [42], Mattsson et al. (1997) [43], Carr et al.
Colon	(2002) [44], Preston et al. (2003, 2007) [45, 46]
Lung	Darby et al. (1994) [47], Preston et al. (2003, 2007) [45, 46]
Basal cell skin carcinoma	Weiss et al. (1994) [48], Carr et al. (2002) [44], Gilbert et al. (2003) [49], Preston et al. (2003, 2007) [45, 46] Schneider et al. (1985) [50], Ron et al. (1991, 1998) [51, 52], Little et al. (1997) [53], Shore et al. (2002)[54], Preston et al. (2007) [46]
Female breast	Howe & McLaughlin (1996) [55], Preston et al. (2002, 2003, 2007) [45, 46, 56]
Thyroid	Lundell et al. (1994) [57], Lindberg et al. (1995) [58], Ron et al. (1995) [59], Preston et al. (2007) [46]
Leukemia, excluding CLL	Little et al. (1999) [60], Travis et al. (2000) [61], Preston et al. (2003, 2004) [45, 62], Muirhead et al. (2009) [63]

Source: Monographs on the evaluation of carcinogenic risks to humans [9]. CLL, chronic lymphocytic leukemia

### Review of epidemiological studies of cancer risk

#### Atomic bomb survivors and the Chernobyl accident

One major source of epidemiological data to evaluate health risks from radiation exposure is the Life Span Study (LSS) of atomic bomb survivors, which found a proportional relationship between cancer risk and exposure dose. Although numerous findings from the study provide scientific evidence for increased cancer risk from radiation exposure, radiation-associated cancer risk remains unclear at low-dose ranges under 100 mSv [11]. Studies related to the Chernobyl accident also demonstrated cancer risks from radiation exposure, especially an increase in thyroid cancer among children with high thyroid-absorbed doses. Except for this result, however, no definitive conclusions have been made regarding other cancers among Chernobyl residents who were exposed to low doses of radiation [12–15]. Some studies that have investigated the health of Chernobyl workers exposed to prolonged low to medium doses of radiation (average effective dose of 100 mSv) have reported increased risks of cancer as well as non-cancer diseases, such as cataracts and cardiovascular diseases [16–21]. However, due to screening effects (e.g., medical examinations) and limited sample sizes, it is difficult to draw definitive conclusions from these studies. Thus, it remains necessary to continue follow-ups of these cohorts with accurate assessments of exposure dose, health outcomes, and confounding factors [14, 22].

#### Occupational exposure in radiation workers

A major distinction between occupational exposure and accidental exposure is the period and dose levels of exposure. Whereas accidental exposure usually involves a single large exposure (acute), occupational exposure involves protracted exposures to low-dose radiation (chronic). A number of epidemiological investigations have been conducted among radiation workers in individual countries as well as in large-scale international cohort studies, and the cancer risk from occupational exposure to radiation continues to be updated. A few studies have reported elevated risks of cancer with statistical significance. One of the largest occupational studies in radiation workers is the 15-country collaborative study, which included 407,391 nuclear industry workers over 5.2 million person-years of follow-up [23]. In this study, an elevated risk of all-cancer mortality was observed, with an excess relative risk (ERR/Sv) of 0.97 (95% CI: 0.27, 1.8). However, this risk diminished after excluding data from workers in Canada, in whom the dose measurement was uncertain, and the observed risk was no longer significant. As a follow-up to the 15-country collaborative study, risks of leukemia and lymphoma were investigated among 308,297 radiation workers in France, the U.K., and the U.S. [24]. The association between exposure dose and cancer mortality was statistically significant with an ERR of 2.96 per Gy (90% CI: 1.17, 5.21) for leukemia, excluding chronic lymphocytic leukemia (CLL). The highest ERR/Gy of 10.45 (90% CI: 4.48, 19.65) was found for chronic myeloid leukemia, indicating a strong association between leukemia mortality and protracted low-dose radiation exposure [24]. Although the ERR of leukemia, excluding CLL, was not attenuated for doses less than 100 mGy, the 90% CIs were too wide to make a definitive conclusion about the low-dose ranges.

Cohort studies of the Mayak nuclear complex workers also reveal an elevated cancer risk [25–27]. Because this cohort had a broad range of cumulative doses due to high exposure levels during the early stages of the facility operation, the dose-response relationship had a degree of precision that is rarely observed in other studies of radiation workers, who are usually exposed to low-dose levels [26]. In addition to the Mayak cohort studies, other studies of radiation workers have reported increased risks of certain types of cancer, such as leukemia (excluding CLL), esophageal cancer, and lung cancer [28–31]. However, risks for individual cancer sites are inconsistent across most radiation epidemiological studies, and many studies do not find statistically significant results. Cancer risks from major health studies in nuclear workers are summarized in Tables 3 and 4.

**Table 3:** Risks of solid cancers in epidemiological studies of nuclear workers

					Mean			Number of event cases		
Country	Study	Cohort size	Exposure period	Follow-up period	cumulative dose (mSv)	Person years	Types of events		ERR (95% CI)	SMR or SIR (95% CI)
15-country	Cardis et al. (2007) [23]	407,391	1943-2000	1943-2000	19.4	5,192,710	Mortality	5,024 4,820	0.97 ^b^(0.27, 1.8)^c^ 0.58 b (-0.1, 1.39)	^a^1.03 (0.65, 1.53)
Korea	^a^Ahn et al. (2008) [64]	79,679	1984-2004 1984-2004	1992-2004 1989-2005	6.1 6.1	633,159 415,298	Mortality Morbidity	256 564	7.2 ^b^(-5, 21) 2.6 (-4, 10)^b^	0.73 (0.64, 0.82)
	Jeong et al. (2010) [65]	8,429	1978-2005	1992-2005	19.86	63,503	Incidence	96	2.06 (-191, 9)	1.06 (0.86, 1.29)
U.K.	Muirhead et al. (2009) [63]	174,541	1946-2001	1965-2001	24.9	3,900,000	Mortality Incidence	7,455 10,855	0.28 (-0.03, 0.62) 0.27 (0.00, 0.56)	0.84 (0.82, 0.86)
U.S.	Howe et al. (2004) [66]	53,698	Mid-1960s	1979-1997	25.7	698,051	Mortality	368	0.51 (-2.01, 4.64)	0.65 (0.59, 0.72)
Canada	Zablotska et al. (2014) [67]	45,316	1951-1994	1956-1994	21.64	613,648	Mortality	468	1.2 (-0.73, 4.33)	0.72 (0.66, 0.78)
France	Flamant et al. (2013) [30]	59,021	1950-2004	1968-2004	16.1	1,467,611	Mortality	2,312	0.34 ^b^(-0.56, 1.38)	-
Germany	Merzenich et al. (2014) [68]	8,972	1966-2008	1991-2008	29.5	130,737	Mortality	119	-	0.63 (0.5, 0.8)
Japan	Akiba et al. (2012) [28]	200,583	1977-2002	1991-2002	12.2	1,373,000	Mortality	2,636	1.26 (-0.27, 3)	-
Russia	Shilnikova et al. (2003) [25]	21,557	1948-1997	1948-1997	810 mGy	720,000	Mortality	1,730	0.15 ^b^(0.09, 0.2)	-
	Hunter et al. (2013) [26]	22,366	1948-2004	1948-2004	510 mGy	535,932	Incidence	1,447	0.07 (0.01, 0.15)	-

^a^ all cancer; ^b^ 90% confidence interval; ^c^ 15-country excluding Canada; ERR, excess relative risk; SMR, standardized mortality ratio; SIR, standardized incidence ratio

**Table 4:** Risks of leukemia (excluding CLL) in epidemiological studies of nuclear workers

					Mean			Number of event cases		
Country	Study	Cohort size	Exposure period	Follow-up period	cumulative dose (mSv)	Person years	Types of events		ERR (95% CI)	SMR or SIR (95% CI)
15-country	Cardis et al. (2007) [23]	407,391	1943-2000	1943-2000	19.4	5,192,710	Mortality	196	1.93 ^b^(<0, 7.14)	-
3-country (INWORKS)	Leuraud et al. (2015) [24]	308,297	1943-2005	1944-2005	15.9mGy	8,220,000	Mortality	531	2.96 (1.17, 5.21)	-
Korea	^a^Ahn et al. (2008) [64]	79,679	1984-2004 1984-2004	1992-2004 1989-2005	6.1 6.1	633.159 415,298	Mortality Morbidity	9 14	16.8 ^b^(-34, 149) 15.8 ^b^(-31, 108)	0.59 (0.28, 1.06)
	Jeong et al. (2010) [65]	8,429	1978-2005	1992-2005	19.86	63,503	Incidence	3	NC	1.34 (0.27, 3.92)
U.K.	Muirhead et al. (2009) [63]	174,541	1946-2001	1965-2001	24.9	3,900,000	Mortality Incidence	198 234	1.71 (-0.17, 4.92) 1.78 (-0.06, 4.99)	0.89 (0.76, 1.03)
U.S.	Howe et al. (2004) [66]	53,698	Mid-1960s	1979-1997	25.7	698,051	Mortality	26	5.67 (-2.56, 30.4)	a 1.07 (0.71, 1.53)
Canada	Zablotska et al. (2014) [67]	45,316	1951-1994	1956-1994	21.64	613,648	Mortality	17	9.79 (<-1.49, 107)	0.78 (0.45, 1.25)
France	Flamant et al. (2013) [30]	59,021	1950-2004	1968-2004	16.1	1,467,611	Mortality	60	3.96 ^b^(<0, 16.82)	-
Germany	Merzenich et al. (2014) [68]	8,972	1966-2008	1991-2008	29.5	130,737	Mortality	7	-	1.19 (0.41, 2.75)
^a^Japan	Akiba et al. (2012) [28]	200,583	1977-2002	1991-2002	12.2	1,373,000	Mortality	80	-1.93 (-6.12, 8.57)	-
Russia	Shilnikova et al. (2003) [25]	21,557	1948-1997	1948-1997	810 mGy	720,000	Mortality	66	1 ^b^(0.5, 2)	-

^a^ all leukemia; ^b^ 90% CI; NC was no convergence of deviance after maximum iteration. CLL, chronic lymphocytic leukemia

Aircrews, such as pilots and flight attendants, are exposed to cosmic radiation. Although aircrews are not included in the national registry for radiation workers in Korea, they should be considered radiation workers and monitored for radiation exposure and health risks, because they are exposed to similar or even higher levels of radiation compared to common radiation-related occupations, such as nuclear workers and radiologists. An average effective dose in an aircrew flying over the poles at high latitudes is estimated to be 2–5 mSv/year, which may reach a cumulative dose of about 75 mSv over the course of a worker’s career [32]. Many interesting health studies have been conducted in aircrews based in Nordic countries, the U.S., and Canada. These studies have reported higher risks of breast cancer, prostate cancer, brain cancer, skin cancer, non-Hodgkin’s lymphoma, and acute myeloid leukemia among aircrews, compared with the general population [33–37]. However, given that no demonstrated dose-response relationship was found, these elevated cancer risks do not imply a causal relationship with radiation exposure.

In summary, despite the existence of several epidemiological studies in radiation workers, cancer risks from occupational exposure, especially for doses less than 100 mSv, remain poorly understood due to uncertainty about exposure dose and confounding factors, possible misclassification of health outcomes, and limited statistical power [24, 38].

### Diagnosed cases of radiation-related occupational cancer in Korea

Recognition of work-related disease is made through the Occupational Disease Approval Committee of the Korea Workers’ Compensation and Welfare Service (COMWEL). According to Article 38 of the Industrial Accident Compensation Insurance Act (IACIA) and Article 7 of the enforcement regulations of the IACIA, the following are diseases that do not require deliberation from COMWEL: (1) pneumoconiosis, (2) carbon disulfide poisoning, (3) diseases with serious acute syndromes from acute exposures to high levels of hazardous agents and relevant risk, and (4) obvious occupation-related disease. In general, criteria for the diagnosis of radiation-related cancers include the cancer site, exposure dose, latent period of cancer, and probability of causation. More strict diagnostic criteria have been applied to thyroid cancer because it is the most common type of cancer found by chance. Table 5 summarizes the characteristics of diagnosed cases of radiation-related occupational cancer in Korea from the occupational disease annual reports (2000–2015) of the Korea Occupational Safety and Health Agency (KOSHA). This list excludes acute diseases due to acute exposure to high levels of hazardous agents and relevant risk according to Article 25 of the enforcement regulations of the IACI Act. Of 43 deliberated cases that may possibly be related to occupational exposure, approximately 70% included male workers, six cases were classified as having a “strong relationship” with occupational exposure, and two cases remained classified as “issues”. All eight cases involved male workers, the youngest of whom was 37 years old. Most of these eight cases had leukemia, including acute myeloid leukemia (AML), acute lymphocytic leukemia (ALL), and chronic myeloid leukemia (CML). Cancers other than leukemia included anaplastic large cell lymphoma, brain cancer, and carcinoma with an unknown primary site.

**Table 5:** Diagnosed cases of radiation-related occupational cancer in Korea (2000~ 2015)

Year	Gender	Age	Occupation	Employment period (year)	Exposure dose (mSv)	Cancer site	Association with occupation
2015	Female	34	Nurse	11.3	Below limits	Breast cancer	Low
	Female	43	Semiconductor manufacturing	7	Below limits	Breast cancer	Low
	Female	42	Semiconductor manufacturing	5.6	0.33	Breast cancer	Low
	Female	35	Semiconductor manufacturing	8.7	Below limits	Breast cancer	Low
	Female	29	Artifact preservation	6.8	Below limits	Intraepithelial carcinoma	Low
	Male	40	Semiconductor manufacturing	5.5	Below limits	Thyroid papillary carcinoma	Low
	Female	33	Semiconductor manufacturing	3.1	Below limits	Brain tumor	Low
2013~ 2014	Male	43	NDT	0.3	7.23	Acute myeloid leukemia	Low
	Male	38	NDT	10	28.84 (for 5 years)	Acute lymphocytic leukemia	High
	Male	47	Radiation oncology specialist	0.8	Possibly over exposure dose limit	Acute lymphocytic leukemia	High
	Male	41	NDT	11	Below limits	Malignant lymphoma	Low
	Male	37	Semiconductor equipment mechanic	13	20.15~ 34.71	Chronic myelomonocytic leukemia	Low
	Male	52	Radiologist	26	Below limits	Rectal cancer	Low
	Female	38	Hospital infection management	11	Below limits	Glioblastoma	Low
	Female	50	Dental nurse	6.4	1.87~ 93.48	Thyroid cancer	Low
	Female	43	Radiologist	18	Below limits	Thyroid cancer	Low
	Male	58	NDT	5	80.77	Aplastic anemia	Low
2012	Male	45	Radiologist	21	204.17	Chronic myeloid leukemia	High
	Male	58	Power plant equipment mechanic	21	1.71	Acute lymphocytic leukemia	Low
	Male	40	X-ray apparatus seller	10.5	140~ 260	Anaplastic large cell lymphoma	High
	Male	53	CT radiographer	18	24.34	Thrombocytopenia	Low
	Male	48	Nuclear worker	7.8	12.25	Stomach cancer	Low
	Female	33	Semiconductor implant operation	4.7	Below limits	Breast cancer	Low
	Male	44	Melting furnace operation	19.6	Below limits	Kidney cancer	Low
2011	Male	42	Artifact preservation	7.2	Below limits	Acute lymphocytic leukemia	Low
	Male	35	Production	1.4	Below limits	Acute myeloid leukemia	Low
2010	Male	39	Machine operator	11	16.51 + potential additional exposure	Acute myeloid leukemia	Issue
	Female	32	Cleaning	5	Below limits	Acute myeloid leukemia	Low
	Male	47	Manufacturing	21	Below limits	Acute myeloid leukemia	Low
	Male	52	Process technician	20	Possible exposure	Brain tumor	Issue
2009	Male	47	Electric power generation worker	21.4	98.32	Stomach and pancreatic cancer cancer	Low
	Male	36	Hospital worker	8	4.5~ 55.4	Thyroid cancer	Low
2008	Female	21	Semiconductor manufacturing	2.5	Below limits	Acute myeloid leukemia	Low
	Male	31	Semiconductor manufacturing	7	Below limits	Acute lymphocytic leukemia	Low
	Female	30	Semiconductor manufacturing	11	Below limits	Acute myeloid leukemia	Low
2005	Male	47	NDT	0.7	Possibly twice over exposure dose limit	Carcinoma of unknown primary site	High
2004	Male	45	Laboratory worker	14	Below limits	Thyroid cancer	Low
	Male	59	Administration	23	51.79	Pancreatic cancer	Low
2002	Male	43	Electric power generation worker	8	1.24	Lung cancer	Low
2001	Male	41	Welder	7	37.87	Non-Hodgkin lymphoma	Low
2000	Male	53	Administration	23	Below limits	Lung cancer	Low
	Male	37	Welder	10	18.5	Acute myeloid leukemia	High
	Male	28	Analyst	2	Below limits	Panmyelophthisis	Low

Below limits: Exposure dose was estimated at natural exposure levels or below the dose limit of radiation workers

*NDT* non-destructive testing, *CT* computed tomography

### Considerations in the recognition criteria for occupational cancer

#### Recognition criteria in Korea

Several criteria should be met cumulatively to obtain the recognition of radiation-related occupational cancer. These criteria are well described in Notification No. 2014-78 of the NSSC regarding regulations on occupational disease among radiation workers. The major criteria are summarized here. First, cancer must be eligible for radiation-induced cancer: liver cancer, except those cancers that involve cirrhosis or the hepatitis virus (e.g., types B or C); thyroid cancer; ovarian cancer; brain cancer; multiple myeloma; colon cancer; bladder cancer; Non-Hodgkin lymphoma; esophagus cancer; kidney cancer; female breast cancer; stomach cancer; pancreatic cancer; salivary gland cancer; lung cancer; skin cancer; and leukemia, excluding CLL. Several cancers are not recognized as radiation-related occupational cancer, namely Hodgkin’s lymphoma, melanoma, malignant mesothelioma, and CLL. These classifications are based mainly on findings from epidemiological studies. For example, mesothelioma is a well-known asbestos-related cancer, and approximately 80–90% of mesotheliomas are caused by long-term inhalation of asbestos [39]. As another example, whereas leukemia is a radiation-sensitive cancer, CLL has not been associated with radiation exposure in most epidemiological studies (Table 6). Second, radiation exposure must be identified by dose assessment or circumstantial evidence. For the assessment of exposure levels, dose records from the NDR are considered a priority. Additional assessments, such as dose reconstruction, are necessary for unclear or omitted cases. Third, a latent period (i.e., time between the first exposure and the appearance of a tumor) must be considered as sufficient or relevant to cancer incidence. For example, solid cancer can be recognized as occupational cancer only if the cancer occurs at least 5 years after the first exposure, whereas leukemia (excluding CLL) can be recognized as occupational cancer only if the cancer occurs at least 2 years after the first exposure and within 20 years after the last exposure. Lastly, the probability of causation (PC), which is defined as the probability that a cancer was caused by occupational radiation exposure during employment, determines whether an individual’s cancer is “at least as likely as not” (i.e., a PC of 50% or greater) related to occupational exposure [40]. The PC is calculated as cancer risk attributable to radiation exposure divided by the sum of baseline cancer risk to the general population plus the risk attributable to radiation exposure, considering personal information (e.g., birth year, gender), medical information (e.g., type of cancer, year of diagnosis), and exposure information (e.g., age at exposure, radiation dose). Given that a threshold dose for cancer has not been identified yet, risks of cancer are stochastic effects, and therefore the PC is an important objective measure to assess a causal relationship with radiation exposure. Based on the current guidelines from the NSSC, PCs for solid cancer and leukemia should exceed 50% and 33%, respectively. However, PC includes an estimation error due to uncertainties about dose and the dose rate effectiveness factor (DDREF), as well as a risk transfer error between different populations; therefore, there exist cases with a PC less than 50% that are fully or partially recognized as occupational cancer in civil litigation.

**Table 6:** Risk of chronic lymphocytic leukemia in epidemiological studies of radiation exposure

Cohort (patients or workers)	Study	Events	Cohort size	Number of events	Risk
Ankylosing spondylitis	Weiss et al. (1995) [69]	Mortality	15,577	7	RR=1.44 (95% CI: 0.62, 2.79)
Benign locomotor lesions	Damber et al. (1995) [70]	Incidence	20,024	50	SIR=1.07 (95% CI: 0.80, 1.41)
Benign gynecological disease	Inskip et al. (1993) [71]	Mortality	12,955	21	RR=1.1 (90% CI: 0.5, 3.0)
Breast cancer	Curtis et al. (1989) [72]	Incidence	22,753	10	RR=1.84 (90% CI: 0.5, 6.7)
Uterine corpus cancer	Curtis et al. (1994) [73]	Incidence	110,000	54	RR=0.90 (95% CI: 0.4, 1.9)
International Radiation	Boice et al. (1988) [42]				OR=1.03 (90% CI: 0.3, 3.9)
Study of Cervical Cancer Patients		Incidence	11,030	52	
Chernobyl liquidators	Romanenko et al. (2008) [74]	Incidence	110,645	39	ERR/Sv=4.09 (95% CI: <0, 14.41)
Chernobyl liquidators	Kesminiene et al. (2008) [20]	Incidence	146,000	21	ERR/Sv=4.7 (90% CI: -®, 76.1)
France nuclear workers	Flamant et al. (2013) [30]	Mortality	59,021	18	ERR/Sv=-1.36 (90% CI: <0, 14.94)
IARC 15-country nuclear workers	Cardis et al. (2007) [23]	Mortality	407,391	47	ERR/Sv=-1.0 (90% CI: -5.0, 3.7)
U.K. NRRW	Muirhead et al. (2009) [63]	Mortality	174,541	69	ERR/Sv=<-1.92 (90% CI: <-1.92, 1.23)
		Incidence	174,541	128	ERR/Sv=-0.117
					(90% CI: -1.42, 2.71)
INWORKS	Leuraud et al. (2015) [24]	Mortality	308,297	138	ERR/Gy=-1.06 (90% CI: <0, 1.81)

RR, relative risk; OR, odds ratio; ERR, excess relative risk; CI, confidence interval; IARC, International Agency for Research on Cancer; NRRW, National Registry for Radiation Workers; INWORKS, International Nuclear Workers Study; ; SIR, standardized incidence ratio

#### Recognition criteria in other countries

The recognition criteria for radiation-related occupational cancer are based on scientific evidence. However, ultimately, their acceptable range and levels are often affected by several factors unrelated to science, such as social, cultural, and economic factors. In particular, complex elements, such as the social status of the radiation-related occupation, number of workers, cancer incidence rate in the general population, specific risk perceptions of certain cancers, and economic wealth, factor into the recognition of occupational cancer. For these reasons, recognition criteria differ across countries or even across occupations within the same country. For example, CLL is generally excluded as an occupational cancer due to lack of scientific evidence regarding radiation-induced CLL. However, CLL is considered as being potentially caused by radiation, and hence, as potentially compensable under the Energy Employees Occupational Illness Compensation Program Act of 2000 (EEOICPA), effective March 7, 2012 in the U.S. In addition, eligible cancer sites differ according to occupation (e.g., special exposure cohort, uranium workers, energy employees, soldiers). Regarding the PC, the EEOICPA applies the upper 99% credibility (i.e., confidence) limit of the PC instead of the point estimate (i.e., 50th percentile) to the determination of causation between exposure and cancer, which provides each worker with the benefit of the doubt before a final compensation decision is made. In France, the criteria for recognition or compensation for cases not relevant to the regulatory guidelines are more relaxed through individual case assessments, meaning that cases with non-radiogenic disease or an inadequate latent period can be possibly compensated when the disease is obviously related to occupational exposure and the disability from the disease is over 25% [41]. Major recognition criteria of Korea and other countries are compared in Table 7.

**Table 7:** Comparison of the recognition criteria of Korea, the U.K., the U.S., and France

Criteria items	Korea	U.K.	U.S. ^a^	France
Eligible cancer sites	Liver (without cirrhosis or hepatitis virus), Thyroid, Ovary, Brain, Multiple myeloma, Colon, Bladder, Non-Hodgkin lymphoma, Esophagus, Kidney, Female breast, Stomach, Pancreas, Salivary gland, Lung, Skin, Leukemia (except CLL)	Bladder, Bone, Brain and central nervous system, Female breast, Colon, Leukemia (except CLL) , Liver, Esophagus, Respiratory/Lung, Prostate, Ovary, Skin (non-melanoma), Uterus, Thyroid, Other tissues	Leukemia with or without CLL, Lymphomas (except Hodgkin lymphomas), Multiple myeloma, Thyroid, Breast, Ovary, Stomach, Lung, Colon, Liver, Bladder, Esophagus, Pancreas, Bone, Salivary gland, Kidney, Brain and central nervous system, Pharynx, Small intestine, Biliary tract and gall bladder, Skin, Rectum, Larynx, Prostate, Pharynx	Leukemia, Primary lung (due to inhalation), Bone sarcoma
Exposure period	-	-	Employed at least 1 year -Uranium miner: >40 months	-
Latency period (since first exposure)	Cancer (except leukemia): 5 years Leukemia (except CLL): 2 years	-	Leukemia (except CLL): 2 years Others: 5 years	-
Occurrence period (after exposure)	Within 20 years	-	Bone cancer: within 30 years Leukemia: any time Others: >5 years	Leukemia and lung cancer: within 30 years Bone sarcoma: within 50 years
PC (Probability of causation) or degree of disability	Cancer (except leukemia): >50% Leukemia (except CLL): >33%	>20% (Compensated at different rates according to the PC and >50% for full compensation)	>50% (upper 99% confidence level)	Degree of disability: >25%
Reference	Notification (No. 2014-78) of the NSSC	Occupational safety and health series 73 [41], Compensation scheme for radiation-linked diseases [75]	Occupational safety and health series 73 [41], Energy employees occupational illness compensation program [76], electronic code of federal regulations [77], radiation exposure compensation Act [78],	Occupational safety and health series 73 [41]

^a^ Eligible cancer sites differ across occupations; exposure period applies only to uranium workers, including uranium miners, millers, ore transporters, and non-military participants in atomic weapons testing; latency period applies only to energy employees employed at the U.S. Department of Energy (DOE) and other specified contractor facilities; occurrence period only applies to soldiers

CLL, chronic lymphocytic leukemia; NSSC, Nuclear Safety and Security Commission

## Conclusions

Based on the scientific evidence and compared with the guidelines of other countries, the current recognition criteria for radiation-related occupational cancer in Korea are valid in terms of the eligibility of cancer sites, adequacy of the latent period, assessment of radiation exposure, and probability of causation. However, the exact quantification of exposure dose is often not possible, and therefore the recognition criteria involve some degree of uncertainty. Therefore, it is proposed that exposure doses of all radiation-related workers be carefully monitored without a dead zone in exposure management, and more relaxed criteria be considered for a margin of uncertainty through the use of the upper 95% or 99% credibility limit of the PC. In addition, further recognition criteria are necessary for more complex exposures, e.g., to two or more carcinogenic agents, including radiation.

## Abbreviations

ALL: Acute lymphocytic leukemia
AML: Acute myeloid leukemia
CAREX: Carcinogen exposure database
CDC: Centers for Disease Control and Prevention
CLL: Chronic lymphocytic leukemia
CML: Chronic myeloid leukemia
COMWEL: Korea Workers’ Compensation and Welfare Service
DDREF: Dose and the dose rate effectiveness factor
EEOICPA: Energy Employees Occupational Illness Compensation Program Act of 2000
ERR: Excess relative risk
IACIA: Industrial Accident Compensation Insurance Act
IARC: International Agency for Research on Cancer
ILO: International Labor Organization
KOSHA: Korea Occupational Safety and Health Agency
NDR: National Dose Registries
NDT: Non-destructive testing
NSSC: Nuclear Safety and Security Commission
NTP: U.S. National Toxicology Program
PC: Probability of causation
